# SETD2-mediated epigenetic regulation of noncanonical Wnt5A during osteoclastogenesis

**DOI:** 10.1186/s13148-021-01125-2

**Published:** 2021-10-18

**Authors:** Moonmoon Deb, Dipranjan Laha, Jyotirindra Maity, Hiranmoy Das

**Affiliations:** Department of Pharmaceutical Sciences, School of Pharmacy, Texas Tech University Health Sciences Center, ARB Suite 2116, 1406 South Coulter Street, Amarillo, TX 79106 USA

**Keywords:** SETD2, Wnt signaling, Osteoclastic differentiation, Epigenetic regulation

## Abstract

To define the role of SETD2 in the WNT5a signaling in the context of osteoclastogenesis, we exploited two different models: in vitro osteoclast differentiation, and K/BxN serum-induced arthritis model. We found that SETD2 and WNT5a were upregulated during osteoclast differentiation and after induction of arthritis. Using gain- and loss-of-function approaches in the myeloid cell, we confirmed that SETD2 regulated the osteoclast markers, and WNT5a via modulating active histone marks by enriching H3K36me3, and by reducing repressive H3K27me3 mark. Additionally, during osteoclastic differentiation, the transcription of *Wnt5a* was also associated with the active histone H3K9 and H4K8 acetylations. Mechanistically, SETD2 directed induction of NF-κβ expression facilitated the recruitment of H3K9Ac and H4K8Ac around the TSS region of the *Wnt5a* gene, thereby, assisting osteoclast differentiation. Together these findings for the first time revealed that SETD2 mediated epigenetic regulation of *Wnt5a* plays a critical role in osteoclastogenesis and induced arthritis.

Model for the Role of SETD2 dependent regulation of osteoclastic differentiation. **A** In monocyte cells SETD2-dependent H3K36 trimethylation help to create open chromatin region along with active enhancer mark, H3K27Ac. This chromatin state facilitated the loss of a suppressive H3K27me3 mark. **B** Additionally, SETD2 mediated induction of NF-κβ expression leads to the recruitment of histone acetyl transferases, P300/PCAF, to the Wnt5a gene and establish H3K9Ac and H4K8Ac marks. Along with other activation marks, these acetylation marks help in Wnt5a transcription which leads to osteoclastogenesis.

**Supplementary Information:**

The online version contains supplementary material available at 10.1186/s13148-021-01125-2.

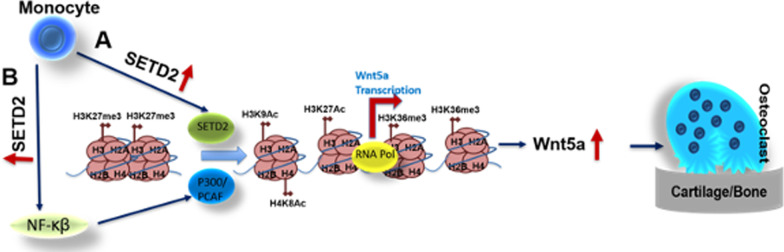

## Introduction

Osteoclasts are large, multinucleated cells of myeloid origin. They are the primary effectors of bone resorption and thus play a critical role in maintaining bone homeostasis; however, dysregulation of these cells causes bone loss in arthritis [1]. Therefore, targeting the differentiation process of osteoclasts might be an effective approach for the prevention and treatment of bone disorders. Osteoclast formation and differentiation are triggered by a series of RANKL and M-CSF-induced signaling events that lead to the activation of transcription factors such as NFATc1 and NF-κB. These transcription factors induce the expression of osteoclast specific genes such as *TRAP*, *CTSK*, and *MMP9* [2, 3].

Epigenetic modifications play critical role in both activation and repression of the gene expressions and histone methylation is one of the most important and complex epigenetic modifications [4]. The histone 3 lysine 36 (H3K36) methyltransferase, which is responsible for tri-methylation of H3K36 (H3K36me3), and is the only histone H3K36-specific methyltransferase identified to date in mammals exists within the SET domain containing 2 (*Setd2*) gene [5–7]. The recruitment of SETD2 is frequently occurred to the sites of active transcription by interaction with RNA polymerase II when it is phosphorylated [8]. The acetylation of H3 on lysine 27 (H3K27Ac), tri-methylation at histone H3 Lys4 (H3K4me3) and H3K36me3 is commonly marked by the chromatin landscape when a gene is actively transcribed. The genetic distribution of the H3K27Ac and H3K4me3 marks are tightly localized around the transcription start site (TSS), however, the distribution of the H3K36me3 modification is mostly in the downstream of the gene, can be throughout the transcribed gene. These H3K27Ac, H3K4me3, and H3K36me3 histone modifications inhibit enzymatic activity through chromatin binding to the polycomb repressive complex 2 (PRC2) [9–11]. In addition, H3K27me3 is preferentially deposited at the CpG-dense promoter region by PRC2 complex, which contain EZH2, H3K27 methyltransferase molecules to repress the transcription of a gene [12].

Wnt signaling has an important role in osteoclastic differentiation. Both hyper and hypoactivation of Wnt signaling results in cartilage breakdown [13]. Wnt3a and Wnt1 are categorized as canonical ligands, whereas and Wnt5a and Wnt11 are believed to be non-canonical ligands. Canonical Wnt signaling suppresses bone resorption and promotes osteoblast differentiation. In contrast, non-canonical Wnt5a-Ror2 signals enhance receptor activator of NF-κB expression in osteoclast precursors by triggering JNK and recruiting c-Jun on the promoter of the RANK gene, thereby enhancing RANK ligand (RANKL)-induced osteoclastogenesis [14]. Several Wnt signaling molecules, such as WNT3A, WNT5A, WNT4, and WNT16 tightly regulate osteoclast differentiation and function to maintain bone mass under physiological conditions [15–17]. However, it is unclear how non-canonical Wnt signaling regulates bone resorption and the fundamental mechanisms involved within.

The importance of SETD2 in osteoclastogenesis and the relation with Wnt signaling remain largely unknown. Herein, we provide evidence that the SETD2 mediated H3K36me3 recruitment plays an essential role in osteoclastic differentiation by modulating the transcriptional initiation and elongation of the Wnt5A. Furthermore, using a K/BxN serum-induced arthritis (RA) model, we shed light on the role of SETD2 in the expression Wnt signaling molecules.

## Results

### Effect of osteoclastic differentiation on SETD2 expression

**Fig. 1:**
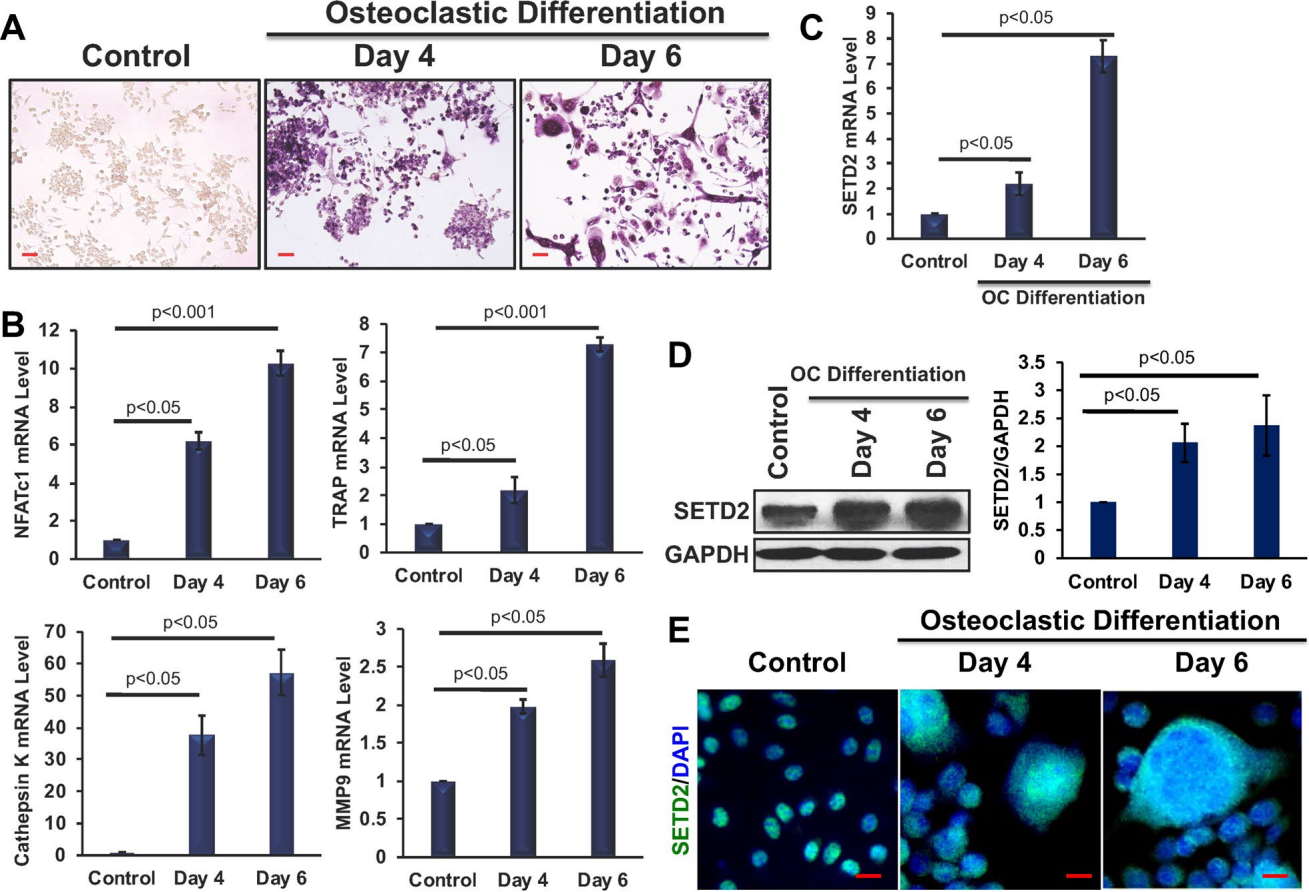
Elevated SETD2 expressions during osteoclastic differentiation. **a** TRAP staining was performed to detect differentiated osteoclasts at days 4 and 6. **b** Relative gene expression of Nfatc1, Trap, Cathepsin K, and Mmp9 were determined in differentiated cells using qRT-PCR keeping β-Actin as an internal control. **c** The relative gene expression of Setd2 was determined in differentiated cells. **d** Protein levels of SETD2 were detected by western blot and the protein expression level was analyzed by ImageJ software. The protein expression level was normalized by using GAPDH expression level and graphically represented. **e** Imunofluorescence analysis of SETD2 expression in osteoclast differentiated samples

To investigate the changes in SETD2 expression levels during osteoclastic differentiation, sRANKL, and MCSF-induced in vitro osteoclastogenesis model was utilized. Faithful osteoclastic differentiation was confirmed by TRAP staining (Fig. 1a), and higher mRNA expression levels of osteoclast-specific genes, such as *Nfatc1, TRAP,* and *Cathepsin K* and osteoclast-associated inflammatory gene, *Mmp9* were observed on days 4 and 6 of osteoclastic differentiation (Fig. 1b). The quantitative RT-PCR results revealed that mRNA levels of *Setd2* were increased 2.1- and 7.2-fold respectively on days 4 and 6 of osteoclast differentiation (Fig. 1c). Additionally, western blot (Fig. 1d) and immunofluorescence staining (Fig. 1e) analysis confirmed the increased levels of SETD2 protein expression after osteoclastic differentiation. These results confirmed that upon induction of osteoclast differentiation, osteoclast cell exhibited upregulation of SETD2 molecule.

### Impact of SETD2 alterations on osteoclast markers

**Fig. 2:**
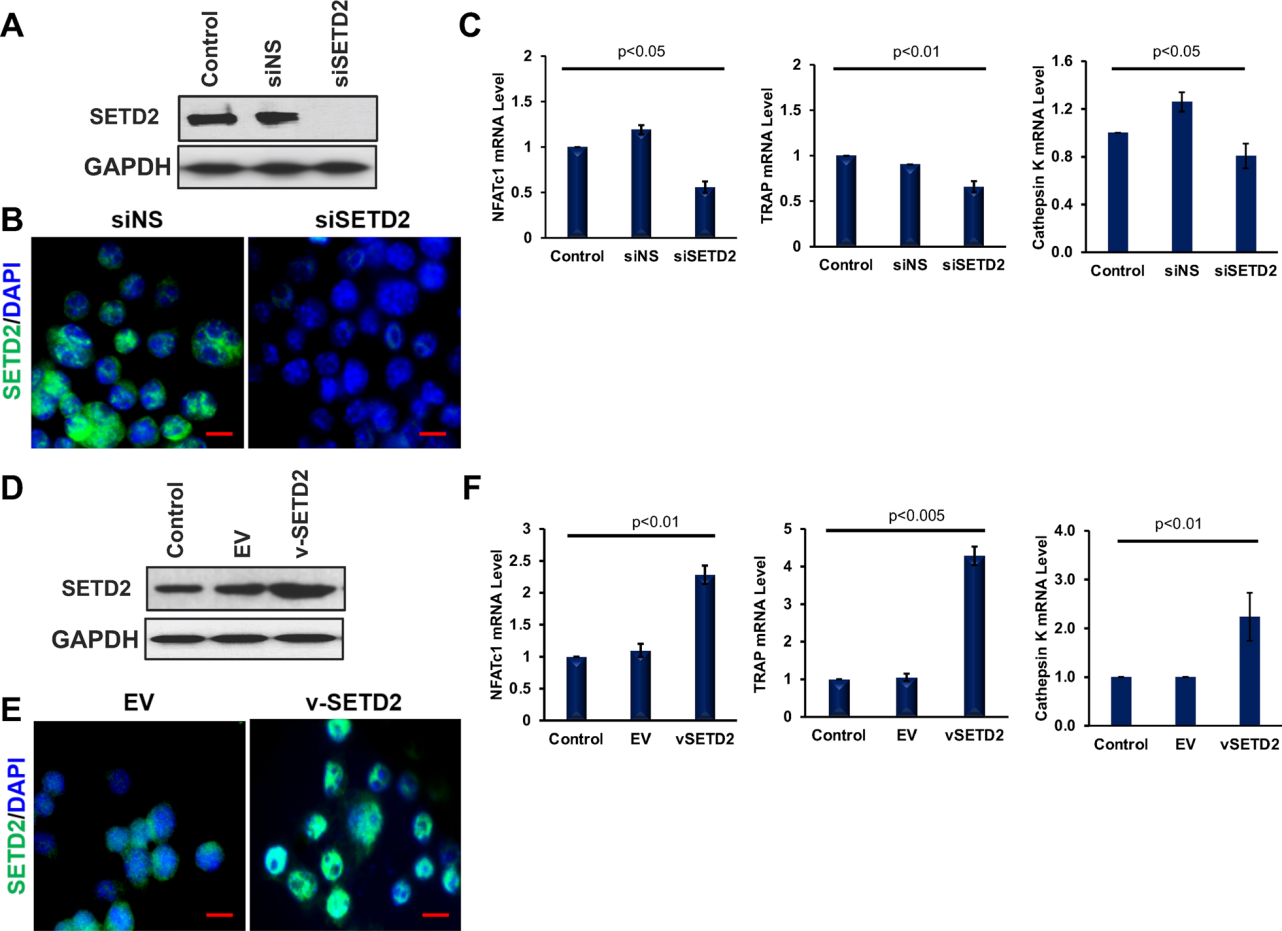
SETD2 induced osteoclast markers expression. Levels of SETD2 was detected by western blot and immunofluorescence after siRNA-mediated knockdown (**a**, **b**, **e**), and overexpression (**c**, **d**, **f**) of SETD2 in RAW264.7 cells. Relative mRNA level expression of Nfatc1, Trap, and Cathepsin K were analyzed by qRT-PCR in SETD2 knocked down and overexpressed samples (**e**, **f**, respectively)

To assess how SETD2 influences the osteoclastogenesis, gain-of-function, and loss-of-function approaches were applied to the osteoclast precursor cells. Western blot and immunofluorescence analyses showed successfully knocked down and overexpressed SETD2 protein (Fig. 2a–d respectively). After establishing the SETD2 upregulation during osteoclastic differentiation; we were inclined to find the influence of SETD2 on osteoclast specific markers such as *Nfatc1*, *Trap* and *Cathepsin K* genes. The qRT-PCR analysis revealed that SETD2 knockdown resulted in a reduced level of *Nfatc1, Trap,* and *Cathepsin K* expression (Fig. 2e). In contrast, the transcriptional activation of *Nfatc1, Trap,* and *Cathepsin K* was enhanced after overexpression of SETD2 molecule (Fig. 2f). These results confirmed that SETD2 can facilitate the transcriptional activation of osteoclast markers in monocytes and thereby effecting osteoclastogenesis process.

### Effect of osteoclast differentiation on Wnt signaling

**Fig. 3:**
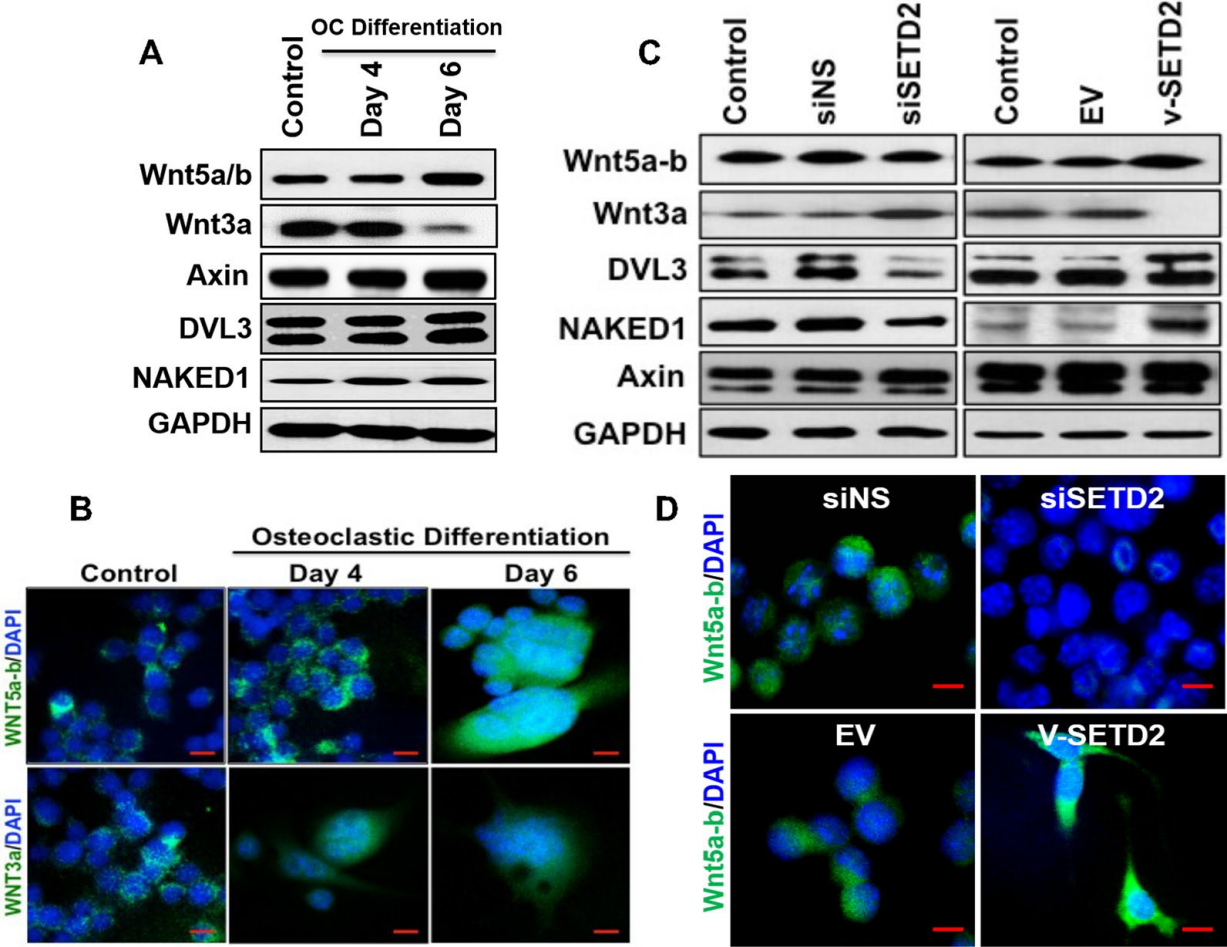
Canonical and non-canonical Wnt pathway molecules during osteoclastic differentiation, and after SETD2 overexpression and knockdown. **a** Western blot detection of Wnt signaling molecules during osteoclastic differentiation of RAW264.7 cells. **b** Detection of WNT5a and WNT3a molecules in cells during osteoclastic differentiation by immunofluorescence method. **C.** Western blot detection of Wnt signaling molecules after knockdown and overexpression of SETD2 in RAW264.7 cells. **d** Detection of WNT5A molecules in cells after overexpression and knockdown of SETD2 by immunofluorescence method. (Scale bar is 10 μM)

We next tested the influence of osteoclast differentiation on the Wnt signaling pathway molecules. To determine the fate of canonical and non-canonical Wnt signaling, we analyzed Wnt molecules in osteoclast differentiated cells. Western blot analysis revealed that non-canonical WNT5a-b protein expression was increased 4.9- and 5.2-fold on day 4 and day 6, respectively, during osteoclastic differentiation compared to undifferentiated cells; conversely, WNT3a expression was remarkably reduced (Fig. 3a, b). We also analyzed the expression level of downstream Wnt pathway molecules that are involved in both canonical and non-Canonical Wnt pathway such as DVL3 and Axin [18, 19]. We found that the expression level of DVL3 was increased with osteoclastic differentiation (Fig. 3a) whereas, Axin remain almost unchanged. For further confirmation about the repression of canonical WNT3a molecule during osteoclastic differentiation, the protein level of NAKED1 (a WNT3a/β-catenin pathway antagonist) was analyzed, and 1.7- and 1.9-fold increase in protein expressions were found on days 4 and 6 of differentiation respectively, compared to undifferentiated cells (Fig. 3a). These observations confirmed that canonical Wnt signaling molecule WNT3a is reduced and non-canonical WNT5a/b is enhanced in differentiated osteoclast cells. This finding indicates the possible involvement of SETD2 in the regulation of the non-canonical WNT5A in myeloid cells.

### Effect of SETD2 modulations on Wnt signaling

After confirming expression profile of the Wnt pathway molecules in myeloid cells, we were inquisitive to identify the effects of SETD2 modulations on canonical and non-canonical Wnt signaling pathway molecules. Based on our data (Figs. 1, 3a) we hypothesized that SETD2 might modulate the Wnt signaling protein expressions during osteoclastic differentiation. To establish this hypothesis, we analyzed WNT5A and WNT3A expressions by western blotting and immunofluorescence staining after SETD2 knockdown or overexpression in RAW 264.7 cells. We found that the SETD2 knockdown resulted in a decreased level of WNT5a expression, and SETD2 overexpression resulted in an increased level of WNT5a expression (Fig. 3c, d).

In contrast, the loss of SETD2 level resulted in increased level of canonical WNT3a expression (Fig. 3c). Whereas, increased SETD2 expression resulted in a decreased expression of WNT3a protein (Fig. 3c). Other Wnt signaling pathway molecules, such as Dvl3 were also decreased with SETD2 knockdown and increase with SETD2 overexpression (Fig. 3c). Levels of Axin remained unchanged after the alteration of SETD2 (Fig. 3c). Interestingly, the canonical Wnt pathway inhibitory molecule, NAKED1 expression was supported by SETD2 overexpression conditions and suppressed in the absence of SETD2. These results suggest that SETD2 can positively regulate non-canonical Wnt signaling molecules, which play a critical role in osteoclastogenesis.

### Transcriptional regulation of Wnt5a by histone modifications during osteoclastic differentiation

**Fig. 4:**
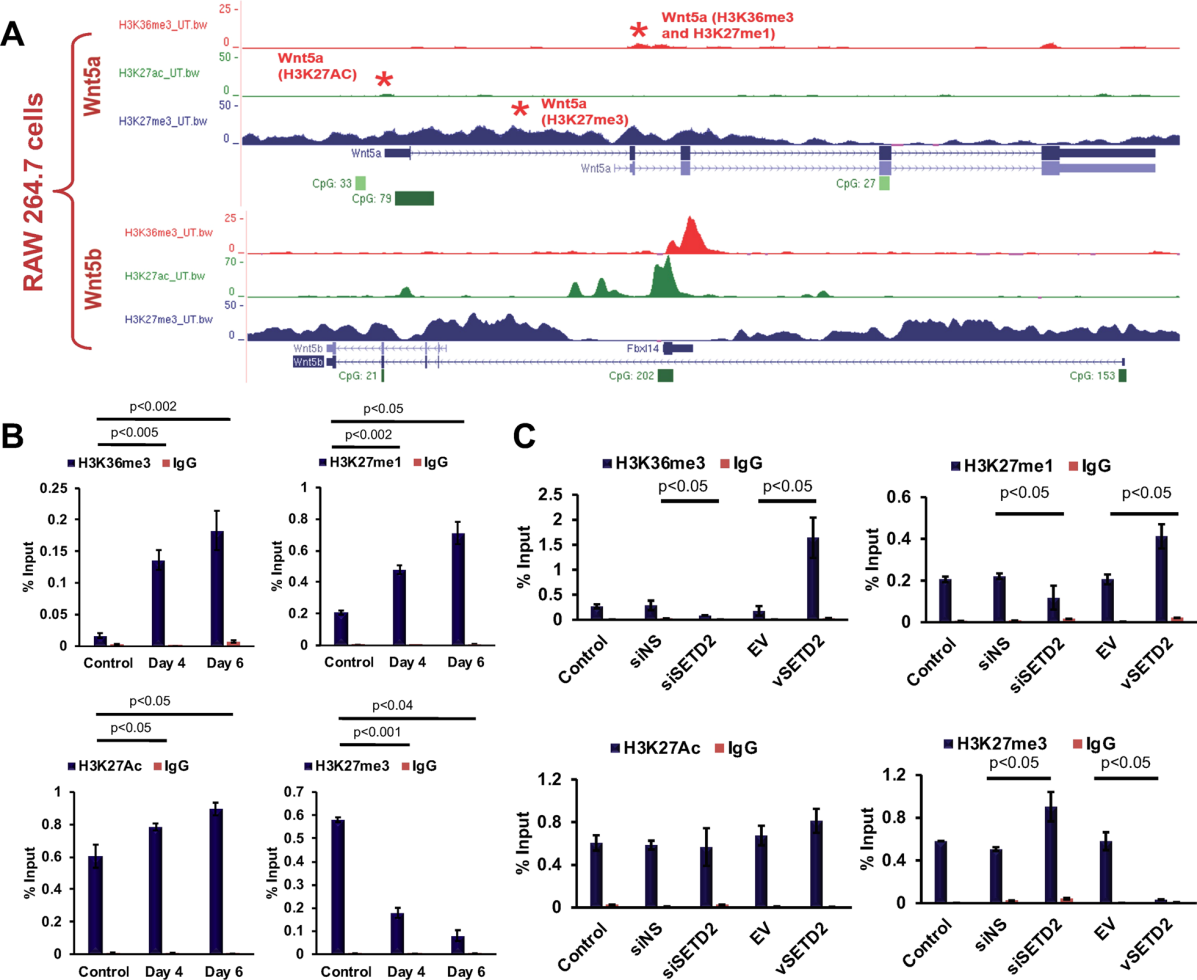
Transcriptional regulations of Wnt5a via SETD2. **a** UCSC genome browser analysis of RAW 264.7 cell line. The image is showing the enrichment pattern of H3K36me3, H3K27Ac, and H3K27me3 marks on the Wnt5a/b gene in wild type untreated RAW 264.7 cells. Red star (*) marks are specific regions that were analyzed by ChIP-qRT-PCR on the Wnt3a gene. **b** Quantitative RT-PCR analysis of ChIP-DNA for H3K36me3, H3K27me1, H3K27me3, and H3K27Ac enrichment on the Wnt5a gene using specific primers designed from the intragenic and upstream region of TSS in osteoclast differentiated cells. **c** ChIP was performed for H3K36me3, H3K27me1, H3K27me3, and H3K27Ac marks on the Wnt5agene in SETD2 knocked down and overexpressed cells. ChIP with IgG rabbit served as a negative control for all ChIP analyses

To our knowledge, no such experimental evidences exist on the transcriptional regulation of non-canonical *Wnt5a* during osteoclast differentiation, or the involvement of SETD2 in this process. UCSC genome browser analysis has shown the enrichment of H3K27me3 mark on *Wnt5a* and *Wnt5b* genes in wild type RAW 264.7 cells (Fig. 4a). This indicates epigenetic control mechanism plays a key role in Wnt5a regulation during osteoclastogenesis. We next sought to evaluate the alteration of histone modifications profile on around the TSS region of *Wnt5a.* After chromatin immunoprecipitation (ChIP), we found that the H3K36me3 and H3K27me1 enrichment on the intragenic region of the *Wnt5a* gene in osteoclast differentiated cells. Additionally, occupancy of active enhancer marks, H3K27Ac, were also elevated around the TSS region of *Wnt5a* (Fig. 4b). In contrast, promoter-H3K27me3 occupancy was significantly decreased (Fig. 4b). H3K36me3, H3K27me1, and H3K27Ac are, however, more strongly enriched in the Day 6 osteoclast differentiated cells than the Day 4 differentiated cells. We believe that this specific arrangement of histone marks on the *Wnt5a* gene could be responsible for the elevated level of *Wnt5a* expression observed during osteoclastic differentiation.

As SETD2 is involved in H3K36me3 recruitment, we postulated, SETD2 may induce transcription of *Wnt5a gene*. Moreover, our experimental data suggested that SETD2 positively regulates WNT5A expression. Based on this evidence we were inclined to find out whether SETD2 may affect the histone modification pattern of *Wnt5a* transcription. To establish the involvement of SETD2 in *Wnt5a* transcription, SETD2 was knocked down and overexpressed in RAW 264.7 cells and subjected to ChIP analysis. The evaluation of histone marks revealed a reduced level of SETD2 expression resulted reducing levels of intragenic, H3K36me3, and H3K27me1 marks on *Wnt5a* gene. In contrast, H3K27me3 occupancy was increased on the *Wnt5a* promoter region (Fig. 4c). Conversely, SETD2 overexpression resulted in elevated levels of H3K36me3 and H3K27me1 marks and loss of the H3K27me3 mark. However, the occupancy of enhancer H3K27Ac mark does not change significantly with the change of SETD2 expression. Though, a slight increase of H3K27Ac was found with the elevated SETD2 expression (Fig. 4c). In conclusion, the transcription of *Wnt5a* is associated with very distinguished histone mark patterns that were identified upstream and within transcribed regions of the *Wnt5a* gene. Verifying this, we confirmed the positive role of SETD2 on the transcription of *Wnt5a* via altering the histone modification profile.

### SETD2 mediated expression of NF-κβ effects on H3K9Ac and H4K8Ac marks on Wnt5a gene

**Fig. 5:**
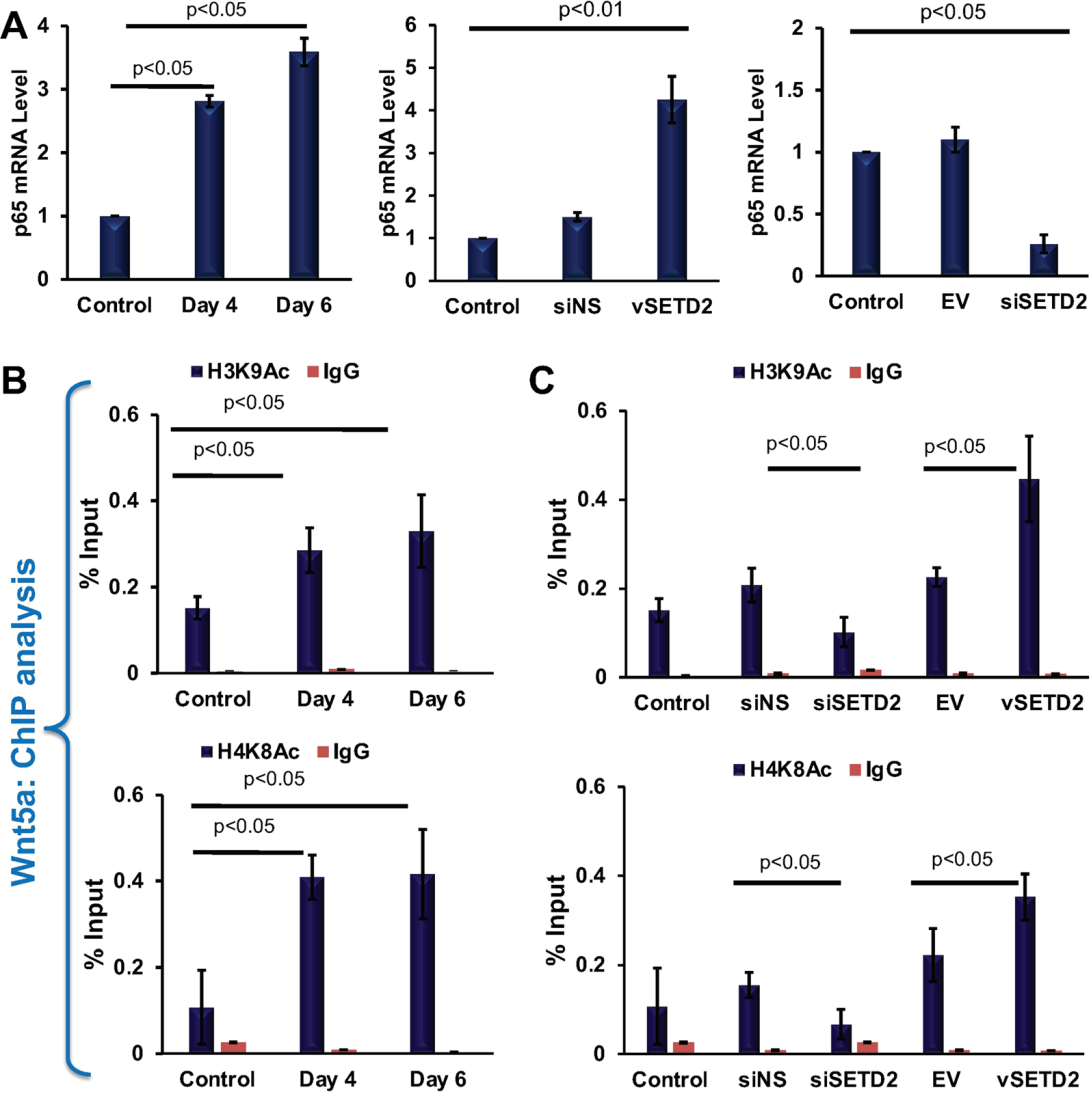
SETD2 facilitated the expression of NF-κβ thus H3K9Ac and H4K8Ac enrichment. **a** RT-PCR analysis of NF-κβ expression in osteoclast differentiated and SETD2 knocked down and overexpressed samples. **b** ChIP-qRT-PCR analysis of H3K9Ac and H4K8Ac enrichment on the Wnt5a gene in osteoclast differentiated and **C.** SETD2 knocked down and overexpressed cells. ChIP with IgG from rabbit served as a negative control for all ChIP analyses

Previous reports indicate that the elevated level H3K9Ac and H4K8Ac facilitated the recruitment of RNA polymerase II and elongation of the osteoclast specific gene such as *Tnfsf11/Rank, Beclin1, Tnf-α* [20]. Moreover, NF-κβ involves in the recruitment of H3K9Ac and H4K8Ac marks [21–23]. Next, we analyzed the p65 (RelA) subunit of NF-κβ expression during osteoclastogenesis and observed the higher level of p65 mRNA expression in osteoclast cells (Fig. 5a). Interestingly, the p65 expression was increased with SETD2 overexpression, and loss of SETD2 led to suppression of P65 transcription (Fig. 5a).

To understand if there is a role of SETD2 in the recruitment of H3K9Ac and H4K8Ac on the *Wnt5a* gene via NF-κβ, we performed the ChIP analysis experiments. The analysis revealed that osteoclastic differentiation significantly increased the active histone marks H3K9Ac and H4K8Ac around the TSS region of *Wnt5a* (Fig. 5b). To further examine whether SETD2 is involved in this regulation, SETD2 overexpress and knocked down samples were also subjected to H3K9Ac and H4K8Ac ChIP analysis. The analysis revealed that the overexpression of SETD2 significantly increased the enrichment of active histone marks H3K9Ac, and H4K8Ac on the *Wnt5a* gene (Fig. 5c). Conversely, SETD2 knock down significantly decreased the H3K9Ac and H4K8Ac occupancy (Fig. 5c). These data suggest that not only H3K36me3 was influenced by SETD2 expression but also SETD2 positively regulated NF-κβ expression thus recruitment of H3K9Ac and H4K8Ac on *Wnt5a* which altogether resulted in WNT5A expression during osteoclast differentiation.

### Effect of K/BxN serum-induced arthritis on SETD2 and Wnt signaling molecules

**Fig. 6:**
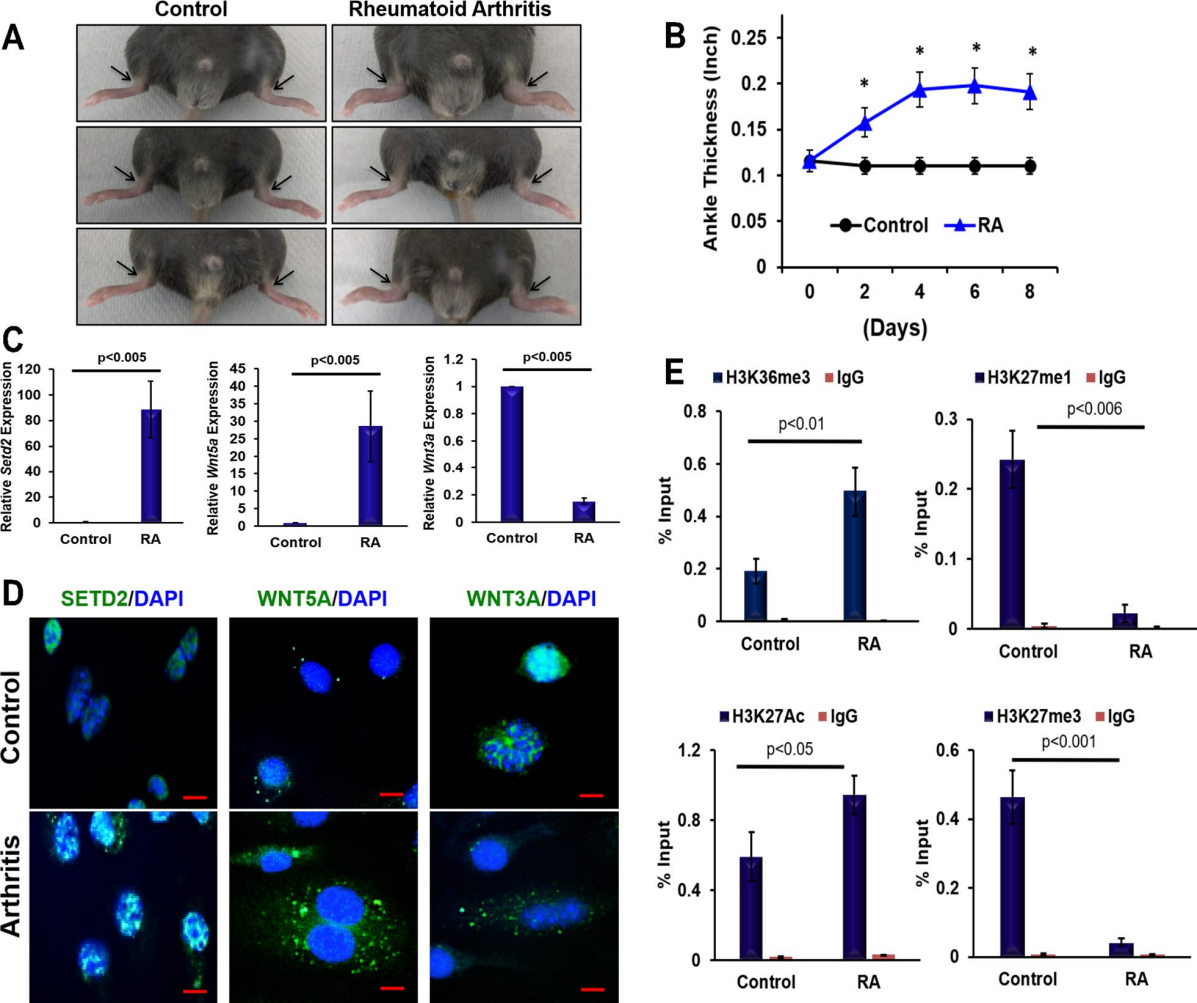
K/BxN serum-induced arthritis effects on SETD2 and Wnt signaling molecules. **a** Ankle morphology (arrowhead) after induced arthritic development. **b** Measured ankle thickness is shown graphically after arthritic development. Star (*) indicates p < 0.05. **c** Relative gene expressions of Setd2, Wnt5a, and Wnt3a were determined in bone marrow monocytes after induction of arthritis in mice using qRT-PCR keeping β-Actin as an internal control. **d** Immunofluorescence analysis of SETD2, WNT5A, and WNT3A protein expression in bone marrow monocytes after arthritic development. **e** ChIP-qRT-PCR analysis of H3K36me3, H3K27me1, H3K27me3, and H3K27Ac enrichment on the Wnt5a gene in bone marrow monocytes after arthritic development. ChIP with IgG rabbit served as a negative control

After in vitro confirmation, we sought to determine whether the mechanistic regulation of SETD2 is persistent in induced arthritis model. The K/BxN serum-induced arthritis (RA) model was established in C57BL6 mice (Fig. 6a). After arthritic inflammation for 8 days, ankle thickness was measured, which showed significantly higher ankle thickness in induced arthritic mice compared to control mice (Fig. 6b). Real-time PCR analysis of bone marrow monocytes revealed that *Setd2* was significantly upregulated in arthritic mice compared to control (Fig. 6c). Additionally, significantly increased levels of *Wnt5a* and decrease the level of *Wnt3a* gene expressions were observed in RA samples (Fig. 6c). As expected, immunofluorescence staining of osteoclastic cells, differentiated from bone marrow precursor cells, revealed higher expression levels of SETD2, WNT5a, but not WNT3a in RA-induced mice compared to controls (Fig. 6d). These observations suggested that SETD2 plays an important role in osteoclastic differentiation as well as in arthritic development, which is associated with non-canonical Wnt signaling.

To determine how *Wnt5a* was transcriptionally regulated, we performed similar ChIP experiments in primary bone marrow cells isolated from control and induced arthritic mice and obtained the similar distribution pattern of all histone marks (Fig. 6e). The analysis confirmed that not only in vitro myeloid cells, but also in vivo primary murine cells transcriptional expression of *Wnt5a* in arthritis associated with enrichment of active marks such as H3K36me3, and H3K27Ac, and reduced levels of H3K27me3 around the TSS region of respective genes (Fig. 6e). Consistent with the previous results, murine data also confirmed that H3K36me3, H3K27me3, H3K27me1, and H3K27Ac marks play significant roles in regulating *Wnt5a* genes during osteoclastic differentiation and arthritis development.

## Discussion

Understanding the mechanisms controlling osteoclast differentiation has enormous clinical importance, especially in arthritis, where hyperactivated osteoclasts are found. Transcriptional activation of osteoclastic markers such as NFATc1, TRAP, and inflammatory genes matrix-metalloproteinase 9, transcription factor, NF-kB, and osteoclastogenesis associated genes receptor, Cathepsin K, are key to myeloid cell activation, osteoclast differentiation, and RA pathogenesis [20, 24–28]. Epigenetic states of a gene are directly related to the transcriptional activation or repression, and modulation in this epigenetic profile or epigenetic modifiers are associated with disease development. Several studies have shown that epigenetic regulations such as genomic DNA methylation, histone acetylation, and miRNA mediated gene silencing contribute to the RA pathogenesis [29–31].

Our study focused on defining the regulation of the Wnt5a molecule via SETD2 the onset of osteoclast differentiation. To our knowledge, this is the first study to show that SETD2 expression is upregulated in osteoclasts (Fig. 1). Here, we used SETD2 loss-of-function and gain-of-function systems to establish the effect of SETD2 on osteoclastogenesis. Our findings of SETD2 mediated increase transcription of osteoclast inducers, NFATc1, TRAP, cathepsin K, provide a clear knowledge about SETD2 induced monocyte activation and osteoclastic differentiation (Fig. 2). Next, we asked how SETD2 affected Wnt signaling onset of osteoclastogenesis. It is well-known that the osteoclast precursors cells provide a suitable microenvironment for osteoclastogenesis by inducing Wnt5a-Ror2 signaling and elevated level of RANK molecules [14, 32, 33]. Additionally, it has been previously reported that Dvl2 and Dvl3 can involve in both canonical and non-canonical Wnt signaling pathways, such as Wnt5a-Ror-Dishevelled signaling [34]. Moreover, Dvl3 is also essential for Wnt5a activation [35]. However, the canonical or non-canonical pathway activation by a given Wnt ligand determined with the cellular context. As an example, non-canonical Wnt5a can both activate and repress Wnt/β-catenin signaling [36]. To resolve this complexity, we have analyzed canonical (Wnt3a), non-canonical (Wnt5a), Wnt signaling associate (DVL3, AXIN), and antagonist of Wnt3a/β-catenin (NAKED1) [37] molecules (Fig. 3). Our results confirm only the activation of the non-canonical Wnt5a pathway not canonical wnt3a signaling, during osteoclastic differentiation. Interestingly, this finding is also strongly supported by the increased expression of canonical Wnt3a pathway inhibitor, Naked1. Similarly, the elevated level of Dvl3 indicated the involvement of the non-canonical WNT5A-DVL3 axis in osteoclastic differentiation.

We hypothesized that SETD2-mediated direct or indirect regulation of WNT5A, in myeloid cells could be the key to osteoclast differentiation. To test our hypothesis, we altered SETD2 expression in myeloid cells and established that SETD2 induced the upregulation of non-canonical WNT5A molecule and impaired canonical Wnt pathway by reducing the WNT3A expression, as well as promoting NAKED1 transcription (Fig. 3). It was reported that WNT3A directly inhibits osteoclast differentiation through both β-catenin and cAMP/PKA pathways [33]. Our data supported a mechanism of SETD2 mediated positive regulation of Wnt5a which facilitated osteoclastogenesis.

Histone lysine methylation and acetylation are two of the most important histone post-translational modifications that regulate chromatin structure. In past decades, the study of SETD2 primarily focuses on their function in cancer biology. Only a few reports have been established in SETD2 related function on bone biology but no data available on SETD2 mediated regulation of osteoclastogenesis. The PRC2 component, PHF19, binds to H3K36me3 and demethylated H3K36me3 through NO66-demethylase to recruit H3K27me3 by EZH2. This model demonstrated that the PRC2 complex uses the unmethylated H3K36 as a signal to recruit H3K27me3 and enrichment of H3K36me3 is inversely related to H3K27me3 level [11]. Our UCSC data analysis has shown that in untreated RAW 264.7 cells H3K36me3 level is low, whereas, H3K27me3 occupancy is high. In our study, we performed ChIP analysis and identified that after osteoclastic differentiation on the gene body region of Wnt5a, H3K36me3 and H3K27me1 occupancies were significantly increased whereas, H3K27me3 level is reduced. Previously reported that the co-existence of H3K27me1 and H3K36me3 can lead to the high mobility of histones and nucleosomes with open chromatin structure to facilitate recruitment of the RNA pol II that eventually lead to transcriptional initiation and elongation which is mutually exclusive with repressive mark, H3K27me3 [38, 39]. Our data indicated that the SETD2-dependent H3K36 trimethylation could induce the transcription of *Wnt5a* during osteoclastogenesis. Additionally, it is also described that global loss of H3K27me3 resulted in accumulation of active enhancer mark, H3K27Ac [40–43]. We observed that the enrichment of H3K27Ac on the Wnt enhancer region, which revealed enhancer activation of the Wnt5a gene during osteoclast differentiation (Fig. 4). The results proved that the co-occurrence of H3K27Ac and H3K36me3 marks and depletion of the H3K27me3 mark facilitated the transcriptional induction of the Wnt5a gene.

Previously described that the presence or absence of H3K9Ac and H4K8Ac marks have a significant role during osteoclastogenesis [25, 27]. H3K9Ac and H4K8Ac facilitated the transcriptional elongation of the osteoclast specific gene such as *Tnfsf11/Rankl* [22] and inflammatory genes such as *Nfkb* and *Tnf-α* [20] by recruiting the RNA Pol II. Keeping this in mind, next we tried to find out whether increased SETD2 expression has any effect on the occupancy of H3K9Ac and H4K8Ac around TSS region of Wnt5a. Our previous study has shown that the transcriptional activity of NF-κβ plays a crucial role in osteoclastogenesis. During monocytic activation, optimal NF-κB-DNA binding requires interaction with several key coactivators such as steroid receptor coactivator (SRC)-1, PCAF, and p300/CBP [20]. Studies revealed that CBP-P300, an H3K9 acetyltransferase [21], and GCN5-PCAF, an H3K9 and H4K8 acetyltransferase [22], are co-existed in most of the promoters and enhancers of NF-κB-regulated genes. It is observed that NF-κB initially binds to the promoter to facilitate the assembly of IRF and ATF-2/c-Jun which serve as a platform for P300 and PCAF recruitment to acetylate the histone molecules [23]. From these studies, it can believe that NF-κB positively regulated the recruitment of H3K9Ac and H4K8Ac marks. We observed that NF-κB (p65) is overexpressed during osteoclast differentiation and increase SETD2 expression is associated with an elevated level of NF-κB expression whereas, SETD2 depletion repress the expression of p65. Next, ChIP data have shown the enrichment of H3K9Ac and H4K8Ac marks around the TSS region of the *Wnt5a* gene in osteoclast differentiated cells. Moreover, SETD2 can positively regulate the level of H3K9Ac and H4K8Ac binding on Wnt5a thus transcription. These data prove that increase SETD2 expression not only influences H3K36me3 enrichment but also induce NF-κB expression which eventually resulted in H3K9Ac and H4K8Ac recruitment and facilitated *Wnt5a* transcription and osteoclast differentiation (Fig. 5).

Increased osteoclast differentiation and activity leading to rapid bone resorption, which caused RA pathogenesis [44]. During RA pathogenesis, monocytes become activated and along with inflammation, osteoclast-mediated cartilage and bone erosion lead to disease development [20, 24–27, 45]. To investigate the association of SETD2 in arthritis, K/BxN serum-induced RA was examined in mice. Our findings revealed that not only osteoclastogenesis, RA development is also associated with increased *Setd2* and non-canonical *Wnt5a* expressions. We also found that enhanced *Wnt5a* expressions are facilitated by enrichment of SETD2 guided active H3H36me3 and enhancer H3K27Ac marks, which facilitated by the reducing level of H3K27me3 mark (Fig. 5). This explains the involvement of SETD2 in RA development and pathogenesis.

In addition, H3K36me3 also participates in cross-talk with other chromatin marks, including DNA methylation. Depending on the cellular micro-environment H3K36me3 may help to established de novo DNA methylation via DNMT3A/B [46] or decreased level of SETD2 can also be associated with increased DNA methylation at intergenic regions in disease condition such as, cancer [47]. The CpG island DNA methylation mainly regulates chromatin accessibility and help to repress transcription of a gene [48].

Along with histone modification DNA methylation also involved in RA pathogenesis. Previously described that gene specific DNA hypomethylation in CD1C gene, and /or hypermethylation in TNFSF10 gene, also involved in RA pathogenesis in human [49]. Our analysis on previously published data sets on human synovial membrane samples [50] confirmed that *Wnt5A* is overexpressed in RA samples (Additional file 1: Figure S1). It was shown that the Wnt signaling plays critical role in immune cell regulation [51–53]. Additionally, DNA methylation data of human RA samples available on epigenome-wide association study (EWAS) datahub established that the level of CpG island DNA methylation was decreased during RA condition on the *Wnt5A* gene (Additional file 2: Figure S2). All together these data indicate that along with rearrangement of histone modification pattern, decreased level of transcription repressor mark, DNA methylation, may have a very important role in the elevated level of *Wnt5a* expression during RA. Based on the previously published and our analyzed data indicate that the SETD2 might be involved in establishing the DNA methylation patten during RA development.

In sum, we found that the SETD2 is one of the key epigenetic regulators of osteoclastogenesis. In this study, we also reveal novel epigenetic mechanisms of non-canonical Wnt5a regulation via SETD2. Enrichment of H3K36me3 and NF-κB arbitrated H3K9Ac and H4K8Ac marks, regulated by SETD2 in monocyte cell to control Wnt5a transcription, thus, involve in osteoclastogenesis and RA development (Fig. 6). Herein, we provide a novel pathway of osteoclastogenesis by SETD2 that sheds light on the interaction of regulatory molecules in the context of RA pathogenesis.

## Materials and methods

### Cell culture and in vitro osteoclastic differentiation

RAW 264.7 cells were cultured in Dulbecco’s modified Eagle’s medium (DMEM, Thermo Fisher Scientific Inc.) supplemented with 10% fetal bovine serum (FBS; Gibco, Life Technologies Corporation) and 100 U/mL PSG at 37 °C with 5% CO_2_. Osteoclast differentiation was performed with earlier established methods [25, 27]. Briefly, sRANKL (40 ng/mL) and M-CSF (20 ng/mL) were added for 4 to 6 days in DMEM supplemented with 10% FBS and 100 U/mL PSG. Fresh medium was replaced every other day along with stimulants and supplements. For, EPZ-6438 treatment**,** RAW 264.7 cells were cultured with sRANKL (40 ng/mL), M-CSF (20 ng/mL) and EPZ-6438 (2.5 nM) for 4 to 6 days in DMEM supplemented with 10% FBS and 100 U/mL PSG. Fresh medium was replaced every other day along with stimulants, supplements, and inhibitor.

### TRAP staining

Differentiated osteoclasts were detected by TRAP staining following the manufacturer's protocol. Briefly, monocytes were cultured on the coverslips in a 6-well plate for differentiation into osteoclasts in the presence of sRANKL (40 ng/mL) and M-CSF (20 ng/mL). On days 4 and 6 of the culture, coverslips were removed from the plate, and cells were fixed for 20 min at room temperature with 4% paraformaldehyde in PBS and then washed with PBS. Next, cells on each coverslip were pre-incubated for 30 s with a mix of 5 μl of sodium nitrite solution and 5 μl Fast Garnet GBC base solution. Cells were then incubated for 1 h at 37 °C in water bath protected from light with a mix of solutions containing 5 μl of acetate, 10 μl of naphthol AS-BI phosphate, 20 μl of tartrate and 450 μl deionized water (pre-warmed to 37 °C) for each well. After incubation, coverslips were rinsed with deionized water thoroughly, mounted on a glass slide, and examined under a light microscope, (Olympus IX81). TRAP-positive cells (purple) containing at least three nuclei were characterized as osteoclast cells.

### SETD2 overexpression and knockdown

Five million RAW 264.7 cells were plated in a 100-mm dish a day prior to over-expression and knockdown experiments. For SETD2 overexpression, cells were transfected with SETD2 containing vector (GFP-SETD2) or empty vector (EV) as a control, using lipofectamine 2000. Conversely, Knockdown of SETD2 was achieved by using ON-TARGETplus Mouse Setd2 siRNA (Dharmacon) keeping non-specific siRNA (NS), as control and transfected with Lipofectamine 2000 (Thermo Fisher Scientific; 11668019). After 48 h of transfection, significant SETD2 overexpression was observed by GFP expression and western blotting. SETD2 knockdown was observed by protein analysis.

### RNA extraction and quantitative RT-PCR

Total RNA was extracted with Trizol reagent (Invitrogen) according to the manufacturer’s instructions. The concentration and quality of the total RNA samples were measured using Nanodrop2000. Complementary DNA was synthesized from 1 µg of total RNA using PrimeScript RT-PCR Kit following the manufacturer’s protocol. RT-qPCR was performed with SYBR-Green RT-PCR Master Mix (Applied Biosystems, Foster City, CA) using the specific primers listed in Additional file 3: Table S1. All mRNA quantities were normalized against β-actin level.

### Immunofluorescence (IF) staining

RAW 264.7 cells (1.0 × 10^5^/well) were seeded on a glass slide placed in a 6-well plate and specific treatment was performed with precise time durations. After treatment, cells were fixed with 4% paraformaldehyde for 15 min and permeabilized with Triton X-100 (0.5%) in PBS for 5 min. Cells were then blocked for 30 min with 5% BSA in PBS and incubated overnight at 4 °C with SETD2, WNT3A, and WNT5A, primary antibodies in 5% BSA. After washing, cells were incubated with anti-rabbit or anti-mouse Alexa Fluor 488 or Alexa Fluor 647 secondary antibody (1:500 dilution; Thermo Fisher Scientific, USA) for 1 h in dark conditions. After incubation, cells were washed thrice with PBS and mounted with 4, 6-diamidino-2-phenylindole, dihydrochloride (DAPI, Invitrogen) on glass slides and sealed with transparent nail varnish. Slides were viewed under a fluorescence microscope and images were captured digitally using an Olympus IX81 microscope with Slide book 5.0 × 64 software.

### Western blotting

For western blotting analysis, the cells were lysed in 100 μl pre-cooled lysis buffer containing 0.5% Triton X100, 150 mM NaCl, and 0.1 U/ml aprotinin in 100 mM Tris–HCl buffer for 30 min on ice and centrifuged at 12,000 × g for 10 min. The concentration of the protein collected in the supernatant was estimated by Bradford's reagent using bovine serum albumin (BSA) as a standard. Equal amounts of proteins (40 μg) were separated in SDS-PAGE gels and transferred to PVDF membranes (Bio-Rad Inc, Irvine, CA). After blocking with 5% BSA for 1 h at room temperature, the membranes were probed with primary antibodies overnight at 4 °C. Antibodies against SETD2 (Cell Signaling Technology, 23,486), Wnt3a (Cell Signaling Technology, 2915), Wnt5a/b (Cell Signaling Technology, 2915), Dvl3 (Cell Signaling Technology, 2915), Naked1 (Cell Signaling Technology, 2915), Axin (Cell Signaling Technology, 2915), and GAPDH (Cell Signaling Technology, 2901), were used. Then membranes were incubated with appropriate horseradish peroxidase (HRP)-labeled secondary antibodies for 2 h at room temperature. Immunoreactive protein bands were visualized by ECL (Amersham Pharmacia Biotech, Piscataway, NJ), and kept the band detections within the linear range. GAPDH was used as an internal control for the whole protein. Developed bands were analyzed by ImageJ software and relative intensity towards the control was reported. Each experiment was repeated 3 times (*n* = 3) and a p value less than 0.05 was considered significant.

### ChIP-qPCR assays

The chromatin immunoprecipitation (ChIP) assays were performed using the Imprint® Chromatin Immunoprecipitation Kit (Sigma) following the manufacturer’s protocol and earlier established protocol [54]. Chromatin was immunoprecipitated against several histone marks, such as H3K36me3, H3K27me1, H3K27me3, H3K4me3, and H3K27Ac. Briefly, isolated RAW 264.7 cells were fixed in 1% formaldehyde, fragmented by sonication. H3K36me3 (Active Motif, 61101), H3K27me3 (Abcam, ab6002), H3K27Ac (Diagenode, C15410174), H3K27me1 (Active Motif, 61015), and negative control IgG (Abcam, ab37373) antibodies were used for immunoprecipitation. After washing and reverse-crosslinking, the precipitated DNA was amplified by primers and quantified by the StepOnePlus real-time-PCR machine (ABI). Primer sequences are provided in the Additional file 4: Table S2. After RT-qPCR, % of input was calculated by using the following formula: ΔCt (normalized ChIP) = [Ct (ChIP)—{Ct (Input)—Log2 (Input Dilution Factor)}]. Where Input Dilution Factor = (Fraction of the input chromatin saved)^−1^. Percent (%) Input = 2 ^(−ΔCt [normalized ChIP])^.

### K/BxN serum-induced arthritis development in mice

To collect the arthritic serum for induction of inflammatory arthritis, K/BxN mice were generated by crossing KRN, TCR-transgenic B6 mice (a kind gift from Dr. Diane Mathis, Harvard Medical School, Boston, MA) with NOD mice (Jackson Laboratory, Bar Harbor, ME) by following established protocol [24, 25, 55]. K/BxN serum was collected from 8-week-old arthritic K/BxN mice and pooled for each experiment. Arthritis was induced in each mouse (*n* = 7, C57BL/6 background 6–8 weeks old, 3 male and 4 female) by intraperitoneal injection of 150 μl of K/BxN serum on days 0 and 2 following an earlier established protocol [56]. Equal amounts of PBS were injected into the control mice (*n* = 7). Ankle thickness was measured every day until sacrifice on day 8 following the first injection of K/BxN serum. Limb bones were used for the isolation of bone marrow, which was used for ChIP analysis, IP assays, and total RNA isolation. TTUHSC IACUC approved all animal experiments. Osteoclast precursors derived from bone marrow were stimulated in triplicate wells with sRANKL (40 ng/mL) and M-CSF (20 ng/mL) for 6 days in DMEM supplemented with 10% FBS and 100 U/mL PSG. Fresh medium was replaced every other day along with stimulants and supplements.

### Statistical analysis

All experiments were performed at least 3 times in triplicate, and the results were displayed as Mean ± SEM. Statistical analyses were performed using Graph Pad Prism 5.0 for Windows (Graph Pad Software, San Diego, CA, USA). Student's t-test was used to perform statistical analysis of RT-qPCR and western blot graph results and p values less than 0.05 were considered significant.

## Supplementary Information

**Additional file 1: Figure S1**. The *Wnt5A* expression in human Synovial membrane samples. Using the previously publish dataset [51], expression pattern of Setd2 and *Wnt5A* was analyzed in 33 in human synovial membrane samples [normal (*N*) = 10, RA (*N*) = 13, osteoarthritis (*N*) = 10]. **A** The detail list of all 33 synovial membrane samples. **B** Graphical representation of *Wnt5A* expression level in normal, RA, and osteoarthritis samples where it shows an elevated expression of Wnt5A in RA samples compared to control samples. [The microarray data from these 33 samples were available as GEO dataset: GDS5403]. **C** Cluster heatmap of the mRNA expression of Setd2 and *Wnt5A* in all samples. Cluster heat map also demonstrate that a higher level of Setd2 and Wnt5A expression in RA samples compared to control samples. Pearson correlation and complete linkage algorithm from GDS cluster analysis software was used to represent the cluster data.

**Additional file 2; Figure S2**. Epigenetic regulation of immune cells is crucial for the development and maintenance of autoimmune diseases like RA. Based on the previous data it showed that B cells are highly relevant to RA pathogenesis, and *Wnt5A-B* signaling is necessary for immune responses [52–54]. DNA methylation profile of the *Wnt5A* gene in RA and control samples in CD19+ B cells, lukocyte and peripheral blood mononuclear cells. Analysis showed that DNA methylation level of *Wnt5A* is decreased in CD19+ B cells of RA compared to healthy samples. [We used EWAS data hub (https://ngdc.cncb.ac.cn/ewas/datahub/index) and CpG probe (cg17246714) to detect DNA methylation level of *Wnt5A* in normal and RA samples].

**Additional file 3: Table S1.** Real-time PCR primers used in determining gene expression.

**Additional file 4: Table S2**. Real-time PCR primer sequences used in ChIP assays.

## References

[1] Firestein GS, McInnes IB (2017). Immunopathogenesis of rheumatoid arthritis. Immunity.

[2] Takayanagi H, Kim S, Koga T, Nishina H, Isshiki M, Yoshida H, Saiura A, Isobe M, Yokochi T, Inoue J, Wagner EF, Mak TW, Kodama T, Taniguchi T (2002). Induction and activation of the transcription factor NFATc1 (NFAT2) integrate RANKL signaling in terminal differentiation of osteoclasts. Dev Cell.

[3] Soysa NS, Alles N (2009). NF-kappaB functions in osteoclasts. Biochem Biophys Res Commun.

[4] Barski A, Cuddapah S, Cui K, Roh TY, Schones DE, Wang Z, Wei G, Chepelev I, Zhao K (2007). High-resolution profiling of histone methylations in the human genome. Cell.

[5] Wagner EJ, Carpenter PB (2012). Understanding the language of Lys36 methylation at histone H3. Nat Rev Mol Cell Biol.

[6] Edmunds JW, Mahadevan LC, Clayton AL (2008). Dynamic histone H3 methylation during gene induction: HYPB/Setd2 mediates all H3K36 trimethylation. EMBO J.

[7] Hu M, Sun XJ, Zhang YL, Kuang Y, Hu CQ, Wu WL, Shen SH, Du TT, Li H, He F, Xiao HS, Wang ZG, Liu TX, Lu H, Huang QH, Chen SJ, Chen Z (2010). Histone H3 lysine 36 methyltransferase Hypb/Setd2 is required for embryonic vascular remodeling. Proc Natl Acad Sci USA.

[8] Kizer KO, Phatnani HP, Shibata Y, Hall H, Greenleaf AL, Strahl BD (2005). A novel domain in Set2 mediates RNA polymerase II interaction and couples histone H3 K36 methylation with transcript elongation. Mol Cell Biol.

[9] Wang Z, Zang C, Rosenfeld JA, Schones DE, Barski A, Cuddapah S, Cui K, Roh TY, Peng W, Zhang MQ, Zhao K (2008). Combinatorial patterns of histone acetylations and methylations in the human genome. Nat Genet.

[10] Kim TW, Kang BH, Jang H, Kwak S, Shin J, Kim H, Lee SE, Lee SM, Lee JH, Kim JH, Kim SY, Cho EJ, Kim JH, Park KS, Che JH, Han DW, Kang MJ, Yi EC, Youn HD (2015). Ctbp2 modulates NuRD-mediated deacetylation of H3K27 and facilitates PRC2-mediated H3K27me3 in active embryonic stem cell genes during exit from pluripotency. Stem Cells.

[11] Brien GL, Gambero G, O'Connell DJ, Jerman E, Turner SA, Egan CM, Dunne EJ, Jurgens MC, Wynne K, Piao L, Lohan AJ, Ferguson N, Shi X, Sinha KM, Loftus BJ, Cagney G, Bracken AP (2012). Polycomb PHF19 binds H3K36me3 and recruits PRC2 and demethylase NO66 to embryonic stem cell genes during differentiation. Nat Struct Mol Biol.

[12] Ferrari KJ, Scelfo A, Jammula S, Cuomo A, Barozzi I, Stutzer A, Fischle W, Bonaldi T, Pasini D (2014). Polycomb-dependent H3K27me1 and H3K27me2 regulate active transcription and enhancer fidelity. Mol Cell.

[13] Nalesso G, Thomas BL, Sherwood JC, Yu J, Addimanda O, Eldridge SE, Thorup AS, Dale L, Schett G, Zwerina J, Eltawil N, Pitzalis C, Dell'Accio F (2017). WNT16 antagonises excessive canonical WNT activation and protects cartilage in osteoarthritis. Ann Rheum Dis.

[14] Maeda K, Kobayashi Y, Udagawa N, Uehara S, Ishihara A, Mizoguchi T, Kikuchi Y, Takada I, Kato S, Kani S, Nishita M, Marumo K, Martin TJ, Minami Y, Takahashi N (2012). Wnt5a-Ror2 signaling between osteoblast-lineage cells and osteoclast precursors enhances osteoclastogenesis. Nat Med.

[15] Kobayashi Y, Thirukonda GJ, Nakamura Y, Koide M, Yamashita T, Uehara S, Kato H, Udagawa N, Takahashi N (2015). Wnt16 regulates osteoclast differentiation in conjunction with Wnt5a. Biochem Biophys Res Commun.

[16] Moverare-Skrtic S, Henning P, Liu X, Nagano K, Saito H, Borjesson AE, Sjogren K, Windahl SH, Farman H, Kindlund B, Engdahl C, Koskela A, Zhang FP, Eriksson EE, Zaman F, Hammarstedt A, Isaksson H, Bally M, Kassem A, Lindholm C, Sandberg O, Aspenberg P, Savendahl L, Feng JQ, Tuckermann J, Tuukkanen J, Poutanen M, Baron R, Lerner UH, Gori F, Ohlsson C (2014). Osteoblast-derived WNT16 represses osteoclastogenesis and prevents cortical bone fragility fractures. Nat Med.

[17] Yu B, Chang J, Liu Y, Li J, Kevork K, Al-Hezaimi K, Graves DT, Park NH, Wang CY (2014). Wnt4 signaling prevents skeletal aging and inflammation by inhibiting nuclear factor-kappaB. Nat Med.

[18] Song XM, Wang S, Li L (2014). New insights into the regulation of Axin function in canonical Wnt signaling pathway. Protein Cell.

[19] Grumolato L, Liu GZ, Mong P, Mudbhary R, Biswas R, Arroyave R, Vijayakumar S, Economides AN, Aaronson SA (2010). Canonical and noncanonical Wnts use a common mechanism to activate completely unrelated coreceptors. Gene Dev.

[20] Das H, Kumar A, Lin Z, Patino WD, Hwang PM, Feinberg MW, Majumder PK, Jain MK (2006). Kruppel-like factor 2 (KLF2) regulates proinflammatory activation of monocytes. Proc Natl Acad Sci USA.

[21] Jin QH, Yu LR, Wang LF, Zhang ZJ, Kasper LH, Lee JE, Wang CC, Brindle PK, Dent SYR, Ge K (2011). Distinct roles of GCN5/PCAF-mediated H3K9ac and CBP/p300-mediated H3K18/27ac in nuclear receptor transactivation. EMBO J.

[22] Huang J, Wan D, Li J, Chen H, Huang K, Zheng L (2015). Histone acetyltransferase PCAF regulates inflammatory molecules in the development of renal injury. Epigenetics.

[23] Bhatt D, Ghosh S (2014). Regulation of the NF-kappa B-mediated transcription of inflammatory genes. Front Immunol.

[24] Das M, Lu J, Joseph M, Aggarwal R, Kanji S, McMichael BK, Lee BS, Agarwal S, Ray-Chaudhury A, Iwenofu OH, Kuppusamy P, Pompili VJ, Jain MK, Das H (2012). Kruppel-like factor 2 (KLF2) regulates monocyte differentiation and functions in mBSA and IL-1beta-induced arthritis. Curr Mol Med.

[25] Das M, Laha D, Kanji S, Joseph M, Aggarwal R, Iwenofu OH, Pompili VJ, Jain MK, Das H (2019). Induction of Kruppel-like factor 2 reduces K/BxN serum-induced arthritis. J Cell Mol Med.

[26] Das M, Deb M, Laha D, Joseph M, Kanji S, Aggarwal R, Iwenofu OH, Pompili VJ, Jarjour W, Das H (2019). Myeloid Kruppel-like factor 2 critically regulates K/BxN serum-induced arthritis. Cells.

[27] Laha D, Deb M, Das H (2019). KLF2 (kruppel-like factor 2 [lung]) regulates osteoclastogenesis by modulating autophagy. Autophagy.

[28] Rolph D, Das H (2020). Transcriptional regulation of osteoclastogenesis: the emerging role of KLF2. Front Immunol.

[29] Araki Y, Mimura T (2017). The histone modification code in the pathogenesis of autoimmune diseases. Mediators Inflamm.

[30] Chen XM, Huang QC, Yang SL, Chu YL, Yan YH, Han L, Huang Y, Huang RY (2015). Role of micro RNAs in the pathogenesis of rheumatoid arthritis: novel perspectives based on review of the literature. Medicine (Baltimore).

[31] Chung YL, Lee MY, Wang AJ, Yao LF (2003). A therapeutic strategy uses histone deacetylase inhibitors to modulate the expression of genes involved in the pathogenesis of rheumatoid arthritis. Mol Ther.

[32] Bellon M, Ko NL, Lee MJ, Yao Y, Waldmann TA, Trepel JB, Nicot C (2013). Adult T-cell leukemia cells overexpress Wnt5a and promote osteoclast differentiation. Blood.

[33] Weivoda MM, Ruan M, Hachfeld CM, Pederson L, Howe A, Davey RA, Zajac JD, Kobayashi Y, Williams BO, Westendorf JJ, Khosla S, Oursler MJ (2016). Wnt signaling inhibits osteoclast differentiation by activating canonical and noncanonical cAMP/PKA pathways. J Bone Miner Res.

[34] Ho HY, Susman MW, Bikoff JB, Ryu YK, Jonas AM, Hu L, Kuruvilla R, Greenberg ME (2012). Wnt5a-Ror-dishevelled signaling constitutes a core developmental pathway that controls tissue morphogenesis. Proc Natl Acad Sci USA.

[35] Ma L, Wang Y, Malbon CC, Wang HY (2010). Dishevelled-3 C-terminal His single amino acid repeats are obligate for Wnt5a activation of non-canonical signaling. J Mol Signal.

[36] Mikels AJ, Nusse R (2006). Purified Wnt5a protein activates or inhibits beta-catenin-TCF signaling depending on receptor context. PLoS Biol.

[37] Van Raay TJ, Fortino NJ, Miller BW, Ma H, Lau G, Li C, Franklin JL, Attisano L, Solnica-Krezel L, Coffey RJ (2011). Naked1 antagonizes Wnt signaling by preventing nuclear accumulation of beta-catenin. PLoS ONE.

[38] Wang LJ, Niu NN, Li L, Sheo R, Ouyang HL, Zou WG (2018). H3K36 trimethylation mediated by SETD2 regulates the fate of bone marrow mesenchymal stem cells. Plos Biol.

[39] Pan MR, Hsu MC, Chen LT, Hung WC (2018). Orchestration of H3K27 methylation: mechanisms and therapeutic implication. Cell Mol Life Sci.

[40] Tie F, Banerjee R, Stratton CA, Prasad-Sinha J, Stepanik V, Zlobin A, Diaz MO, Scacheri PC, Harte PJ (2009). CBP-mediated acetylation of histone H3 lysine 27 antagonizes Drosophila Polycomb silencing. Development.

[41] Creyghton MP, Cheng AW, Welstead GG, Kooistra T, Carey BW, Steine EJ, Hanna J, Lodato MA, Frampton GM, Sharp PA, Boyer LA, Young RA, Jaenisch R (2010). Histone H3K27ac separates active from poised enhancers and predicts developmental state. Proc Natl Acad Sci USA.

[42] Pradeepa MM, Grimes GR, Kumar Y, Olley G, Taylor GC, Schneider R, Bickmore WA (2016). Histone H3 globular domain acetylation identifies a new class of enhancers. Nat Genet.

[43] Heintzman ND, Stuart RK, Hon G, Fu YT, Ching CW, Hawkins RD, Barrera LO, Van Calcar S, Qu CX, Ching KA, Wang W, Weng ZP, Green RD, Crawford GE, Ren B (2007). Distinct and predictive chromatin signatures of transcriptional promoters and enhancers in the human genome. Nat Genet.

[44] McHugh J (2020). Precursors of 'bad' osteoclasts identified in arthritis. Nat Rev Rheumatol.

[45] Rolph DN, Deb M, Kanji S, Greene CJ, Das M, Joseph M, Aggarwal R, Leblebicioglu B, Das H (2018). Ferutinin directs dental pulp-derived stem cells towards the osteogenic lineage by epigenetically regulating canonical Wnt signaling. Biochim Biophys Acta Mol Basis Dis.

[46] Xu Q, Xiang Y, Wang Q, Wang L, Brind'Amour J, Bogutz AB, Zhang Y, Zhang B, Yu G, Xia W, Du Z, Huang C, Ma J, Zheng H, Li Y, Liu C, Walker CL, Jonasch E, Lefebvre L, Wu M, Lorincz MC, Li W, Li L, Xie W (2019). SETD2 regulates the maternal epigenome, genomic imprinting and embryonic development. Nat Genet.

[47] Tiedemann RL, Hlady RA, Hanavan PD, Lake DF, Tibes R, Lee JH, Choi JH, Ho TH, Robertson KD (2016). Dynamic reprogramming of DNA methylation in SETD2-deregulated renal cell carcinoma. Oncotarget.

[48] Dahlet T, Argueso Lleida A, Al Adhami H, Dumas M, Bender A, Ngondo RP, Tanguy M, Vallet J, Auclair G, Bardet AF, Weber M (2020). Genome-wide analysis in the mouse embryo reveals the importance of DNA methylation for transcription integrity. Nat Commun.

[49] Julia A, Absher D, Lopez-Lasanta M, Palau N, Pluma A, WaiteJones L, Glossop JR, Farrell WE, Myers RM, Marsal S (2017). Epigenome-wide association study of rheumatoid arthritis identifies differentially methylated loci in B cells. Hum Mol Genet.

[50] Woetzel D, Huber R, Kupfer P, Pohlers D, Pfaff M, Driesch D, Haupl T, Koczan D, Stiehl P, Guthke R, Kinne RW (2014). Identification of rheumatoid arthritis and osteoarthritis patients by transcriptome-based rule set generation. Arthritis Res Ther.

[51] MastelarodeRezende M, ZenkerJusto G, JulianParedes-Gamero E, Gosens R (2020). Wnt-5A/B signaling in hematopoiesis throughout life. Cells.

[52] Jati S, Sarraf TR, Naskar D, Sen M (2019). Wnt signaling: pathogen incursion and immune defense. Front Immunol.

[53] Mehmeti M, Bergenfelz C, Kallberg E, Millrud CR, Bjork P, Ivars F, Johansson-Lindbom B, Kjellstrom S, Andre I, Leandersson K (2019). Wnt5a is a TLR2/4-ligand that induces tolerance in human myeloid cells. Commun Biol.

[54] Maity J, Deb M, Greene C, Das H (2020). KLF2 regulates dental pulp-derived stem cell differentiation through the induction of mitophagy and altering mitochondrial metabolism. Redox Biol.

[55] Kouskoff V, Korganow AS, Duchatelle V, Degott C, Benoist C, Mathis D (1996). Organ-specific disease provoked by systemic autoimmunity. Cell.

[56] Jacobs JP, Ortiz-Lopez A, Campbell JJ, Gerard CJ, Mathis D, Benoist C (2010). Deficiency of CXCR2, but not other chemokine receptors, attenuates autoantibody-mediated arthritis in a murine model. Arthritis Rheum.

