# Supplementary figures and images for: SETD2-mediated epigenetic regulation of noncanonical Wnt5A during osteoclastogenesis
Source: Clin Epigenetics. 2021 Oct 18;13:192. doi: 10.1186/s13148-021-01125-2 (PMC8522097; doi:10.1186/s13148-021-01125-2)

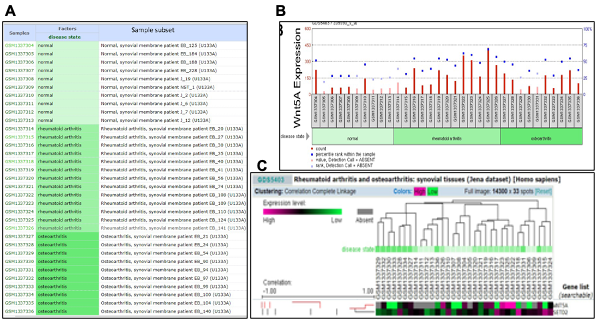

Supplement: Supplementary file 1 — Additional file 1: Figure S1. The Wnt5A expression in human Synovial membrane samples. Using the previously publish dataset [51], expression pattern of Setd2 and Wnt5A was analyzed in 33 in human synovial membrane samples [normal (N) = 10, RA (N) = 13, osteoarthritis (N) = 10]. A The detail list of all 33 synovial membrane samples. B Graphical representation of Wnt5A expression level in normal, RA, and osteoarthritis samples where it shows an elevated expression of Wnt5A in RA samples compared to control samples. [The microarray data from these 33 samples were available as GEO dataset: GDS5403]. C Cluster heatmap of the mRNA expression of Setd2 and Wnt5A in all samples. Cluster heat map also demonstrate that a higher level of Setd2 and Wnt5A expression in RA samples compared to control samples. Pearson correlation and complete linkage algorithm from GDS cluster analysis software was used to represent the cluster data. [file 13148_2021_1125_MOESM1_ESM.tiff]

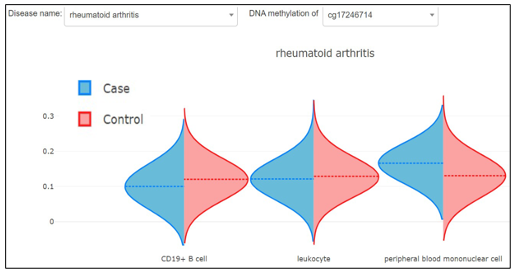

Supplement: Supplementary file 2 — Additional file 2; Figure S2. Epigenetic regulation of immune cells is crucial for the development and maintenance of autoimmune diseases like RA. Based on the previous data it showed that B cells are highly relevant to RA pathogenesis, and Wnt5A-B signaling is necessary for immune responses [52–54]. DNA methylation profile of the Wnt5A gene in RA and control samples in CD19+ B cells, lukocyte and peripheral blood mononuclear cells. Analysis showed that DNA methylation level of Wnt5A is decreased in CD19+ B cells of RA compared to healthy samples. [We used EWAS data hub (https://ngdc.cncb.ac.cn/ewas/datahub/index) and CpG probe (cg17246714) to detect DNA methylation level of Wnt5A in normal and RA samples]. [file 13148_2021_1125_MOESM2_ESM.tiff]

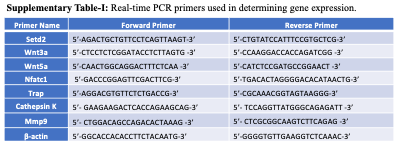

Supplement: Supplementary file 3 — Additional file 3: Table S1. Real-time PCR primers used in determining gene expression. [file 13148_2021_1125_MOESM3_ESM.tiff]

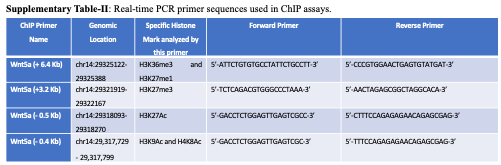

Supplement: Supplementary file 4 — Additional file 4: Table S2. Real-time PCR primer sequences used in ChIP assays. [file 13148_2021_1125_MOESM4_ESM.tiff]
